# Joint association of physical activity and overweight with subsequent psychotropic medication: a register-linked follow-up study among employees

**DOI:** 10.1186/s12889-015-2346-5

**Published:** 2015-10-03

**Authors:** Tiina Loponen, Tea Lallukka, Ansku Holstila, Jouni Lahti

**Affiliations:** Department of Public Health, University of Helsinki, Helsinki, Finland; Centre of Expertise for Health and Work Ability & Disability Prevention Research Centre, Finnish Institute of Occupational Health, Helsinki, Finland

**Keywords:** Body mass index, Physical activity, Register-linked, Cohort, Mental health

## Abstract

**Background:**

Physical activity level and overweight have shown associations with mental health problems but it is not known whether the risk of mental health problems due to overweight varies by physical activity. We examined joint association of physical activity and overweight with subsequent psychotropic medication among 40–60-year-old employees.

**Methods:**

The questionnaire survey data were derived from Helsinki Health Study baseline postal questionnaires in 2000–02 among employees of the City of Helsinki aged 40–60 years (*n* = 8960, response rate 67 %). Baseline survey data were linked with prospective register data on prescribed psychotropic medication (ATC-codes N05 and N06, except N06D) among those with written consent (74 %) for such linkage. The analyses included 6169 responders (78 % women, corresponding to the target population). We divided participants into six groups according to their baseline self-reported body mass index and leisure-time physical activity using physically highly active normal-weight participants as a reference group. We used Cox regression analysis adjusted for age, gender, psychotropic medication prior to baseline, and socioeconomic position, marital status, working conditions, limiting long-standing illness, alcohol use, and smoking.

**Results:**

At baseline, 49 % were overweight and 23 % were physically inactive. After adjusting for age and gender, inactive normal-weight (hazard ratio (HR) 1.3, 95 % CI 1.1–1.5), moderately active overweight (HR 1.3, 95 % CI 1.1–1.5) and inactive overweight (HR 1.4, 95 % CI 1.2–1.6) had higher risk for any psychotropic medication compared with group of highly active normal-weight. After adjusting for prior medication, only the inactive overweight group had higher risk (HR 1.4, 95 % CI 1.2–1.6). Other covariates made but a minor contribution to the examined associations. For antidepressants the associations were somewhat stronger than for sedatives.

**Conclusions:**

Both normal-weight and physical activity help prevent psychotropic medication but physical activity dominates the association over normal-weight.

## Background

Mental health problems are increasing around the world and are topical issues from public health perspective [1] and better treatment might be beneficial to decreasing costs of mental health problems. Psychotropic medication is widely used in general population of various countries [2–4] and there is evidence that prescribed psychotropic medication is more common among women than men [3, 4]. Poor mental health further is a major cause for work disability and disability-retirement [5].

Physical inactivity and overweight are associated with mental health problems [6–9]. Additionally, physical inactivity alone is a public health risk factor and a number of studies have showed that leisure-time physical inactivity increases the risk of various diseases and mortality [10–13]. Leisure-time physical activity may also be important in reducing the risk of mental health problems [14] and even small amounts of physical activity may protect against depression [7]. Furthermore, some studies suggest that higher amounts of physical activity may show further benefits for mental health [6, 15]. Physical activity has been successfully used to treat depression [16–18] and some anxiety disorders [16] although the evidence is still weak. A study of six European populations [8] showed that obese people had more mood disorders and comorbid mental disorders compared to normal-weight population. The association between high BMI and mental health problems is seen clearest in post-traumatic stress disorder and in different anxiety disorders [9].

Earlier longitudinal study from the same data showed that physical activity is likely to be beneficial for self-reported mental health functioning among both normal-weight and overweight adults after a follow-up of 5 to 7 years [19]. Furthermore, a cross-sectional study of physical activity and overweight with health-related quality of life showed that physical activity might be more important than normal-weight for health-related quality of life [20]. We still lack studies of joint-association of physical activity and overweight with subsequent mental health using objective register based measures such as psychotropic medication.

The aim of this study was to examine joint association of leisure-time physical activity and overweight with subsequent psychotropic medication. The contribution of prior medication and other key covariates such as smoking, drinking problems, limiting long-standing illness, socioeconomic position, employment status, and marital status to the association was also examined, because they have been associated with mental health in previous studies [21–24]. We expected that normal-weight and high physical activity is the best protection against mental health problems and psychotropic medication. Based on previous study [6] we further expected that high physical activity might protect against psychotropic medication also among the overweight.

## Methods

The questionnaire survey data were derived from the Helsinki Health Study baseline postal questionnaires in 2000–02. Participants were employees of the City of Helsinki, Finland, aged 40–60 years. There were 8960 responders (response rate 67 %) of whom 78 % were women. The response rate was somewhat lower among men, in younger age groups, lower occupational classes and those with long sickness absence. The data were linked to the register of prescribed psychotropic medication from the Social Insurance Institution of Finland. Seventy-four percent of participants gave permission for linkages and non-response analysis showed that non-response and non-consent unlikely biased results [25, 26]. The follow-up started at the day of returning the questionnaire and ended to first psychotropic medication purchase or death. The maximum follow-up time was 7.8 years and mean follow-up time was 6.4 years (SD 2.45). Those with psychotropic medication at baseline (*n* = 319) or with missing information on leisure time physical activity (*n* = 43), BMI (*n* = 47) or other covariates (*n* = 27) were excluded. The analyses included 6169 responders and 78 % on those were women. The ethics committees of the Department of Public Health, University of Helsinki and health authorities of the City of Helsinki have approved the Helsinki Health Study.

### Physical activity

The participants were asked about the average weekly time they spent in leisure-time physical activity (commuting included) within the last 12 months. They were first asked to estimate the intensity of their physical activity compared to common activities: walking, brisk walking, jogging, and running, or activities equivalent to these. After that they were asked to estimate on average how much time they spent in each intensity grade in 1 week. Based on responds of leisure-time physical activity, an approximate metabolic equivalent (MET) value was calculated [27]. The average MET hours of leisure-time physical activity in 1 week were calculated by multiplying time used weekly by the estimated MET value of the each intensity grade [28] and then summing all four MET values together. The participants were then grouped into inactive, moderately active, and highly active groups and as cut-off points we used the recommended levels of physical activity [29]. The physically inactive group did under 14 MET hours weekly and it corresponds approximately to 1000 kcal per week or less. Moderately active group did 14–30 MET hours per week (approximately 1000–2000 kcal). Highly active group did over 30 MET hours (approximately over 2000 kcal) of physical activity per week, which is the recommended level for healthy weight maintenance [29].

### Body mass index

Body mass index was calculated from self-reported weight (kg) and height (m). Participants whose BMI was under or equal to 25 kg/m^2^ were grouped as normal-weight and whose BMI was over 25 kg/m^2^ were grouped as overweight. Ca 1 % (*n* = 59) of participants were underweight (BMI under 18,5 kg/m^2^) and those were excluded from the sample but no effect to the association was found and underweight participants were retained in the final sample.

### Psychotropic medication

Psychotropic medication data from Social Insurance Institution of Finland were classified according to the Anatomical Therapeutic Chemical (ATC) classification system [30]. ATC codes N05 and N06 (except N06D which is medication for dementia) were included to any psychotropic medication. We also examined antidepressants (N06A) and anxiolytics and sedatives (N05B and N05C) separately but groups were not mutually exclusive. The follow up started at the day of returning the questionnaire and ended to first psychotropic medication purchase or death. Mortality data were derived from the registers of Statistics Finland.

### Covariates

Covariates derived from the baseline questionnaire included age, gender, marital status, physical and mental strenuousness of work, smoking, drinking problems, and limiting long-standing illness (LLI). Socioeconomic position was derived from the employer’s registers and prior psychotropic medication was derived from the register of Social Insurance Institution of Finland. At baseline age was classified into five groups: 40, 45, 50, 55 and 60 years. Information on prior psychotropic medication 3 years preceding baseline were included. Socioeconomic position was measured by four occupational classes: managers/professionals, semi-professionals, routine non-manual employees, and manual workers [31]. Marital status was dichotomized to partnership or no partnership. Physical strenuousness of work was dichotomized to light or heavy. Similar procedure was followed for mental strenuousness of work. Smoking was divided to smokers and non-smokers. Alcohol use was measured by CAGE questionnaire, which describes drinking problems [32]. LLI was dichotomized into those reporting any limiting long-standing illness and those not reporting LLI.

### Statistical methods

We divided participants into six groups according to their body mass index and leisure-time physical activity: (1) inactive (under 14 METs) and normal-weight (BMI ≤ 25 kg/m^2^), (2) moderately active (14–30 METs) normal-weight, (3) highly active (over 30 METs) normal-weight, (4) inactive overweight (BMI > 25 kg/m^2^), (5) moderately active overweight, and (6) highly active overweight. The group of active normal-weight participants was used as a reference group in all the analyses. Proportions and 95 % confidence intervals for subsequent psychotropic medication was first calculated. Cox regression analysis was then used to examine the effect of covariates and to calculate hazard ratios (HR) and their 95 % confidence intervals (95 % CI). Men and women were in the pooled data adjusting for gender because there was no significant difference between genders (*p* = 0.44 for interaction). In model 1 age and gender were adjusted for. In addition to covariates in model 1, in model 2 prior medication was adjusted for. In model 3 covariates in model 2 and socioeconomic position, marital status, and physical and mental strenuousness at work were adjusted for. In model 4 covariates in model 2 and smoking, drinking problems, and LLI were adjusted for. We used IBM SPSS Statistics version 22.0 for mac.

## Results

At baseline, 19 % were physically highly active and normal-weight and 18 % were physically inactive and overweight (Table 1). During the follow-up, 30 % of participants had at least one reimbursed purchase of any psychotropic medication. Every fifth had antidepressant purchases and only slightly fewer had sedative purchases. Thirty-two percent of those who had any purchases of psychotropic medication purchased both sedatives and antidepressants. Exclusively antidepressant purchases were 35 % and exclusively sedatives 29 % of all psychotropic medication purchases. Nineteen percent of participants had had psychotropic medication within the last 3 years before the baseline survey.

**Table 1:** Baseline participant characteristics

	Men (%/n)	Women (%/n)	All (%/n)
	% (n)	% (n)	% (n)
Age (mean)	50.1	49.2	49.4
Overweight (BMI > 25 kg/m^2^)	60.3	45.7	48.8
Activity groups			
Highly active	41.8	36.9	38.0
Moderately active	33.3	40.1	38.6
Inactive	25.0	22.9	23.4
Weight/activity groups			
Highly active normal-weight	19.1	23.6	22.6
Moderately active normal-weight	13.2	21.1	19.4
Inactive normal-weight	7.4	9.6	9.1
Highly active overweight	22.7	13.3	15.4
Moderately active overweight	20.0	19.0	19.2
Inactive overweight	17.6	13.4	14.3
Medication during follow-up			
Any	22.8	31.6	29.7
Antidepressant	15.6	21.3	20.1
Sedatives	14.6	19.3	18.3
Prior medication			
Any	14.0	20.9	19.4
Antidepressant	6.7	10.5	9.6
Sedatives	8.7	13.0	12.1
Work physical strenousness			
Light	85.2	62.1	67.1
Heavy	14.8	37.9	32.9
Work mental strenousness			
Light	85.4	86.6	86.3
Heavy	14.6	13.4	13.7
Marital status (in a relationship)	78.4	69.0	71.1
Sosioeconomic position			
Manual worker	25.9	11.1	14.3
Routine non-manual	9.4	41.2	34.3
Semi-professional	19.9	19.4	19.5
Managers/professionals	44.8	28.4	32.0
Limiting long-standing illness (LLI)	16.2	16.3	16.3
Drinking problems (cage)	23.3	15.2	16.9
Smoking	25.4	21.6	22.4
n	21.7 (1338)	78.3 (4831)	100 (6169)

Psychotropic medication tended to be more common in physically inactive overweight (34 %, 95 % CI 30.4–36.6) and normal-weight (33 %, 95 % CI 29.3–37.1) than in the highly active overweight (25 %, 95 % CI 22.6–28.1) and normal-weight (27 %, 95 % CI 24.7–29.3) groups (Table 2). The patterns were similar for antidepressants and sedatives.

**Table 2:** Prevalence of first psychotropic medication purchases (%) during the follow-up

	n	Any (%)	Antidepressants (%)	Sedatives (%)
All	6169	29.7 (28.6–30.9)	20.1 (19.1–21.1)	18.3 (17.3–19.3)
Highly active normal-weight	1397	27.0 (24.7–29.3)	17.3 (15.3–19.2)	16.3 (14.4–18.3)
Moderately active normal-weight	1198	30.4 (27.8–33.0)	20.2 (17.9–22.5)	19.5 (17.3–21.8)
Inactive normal-weight	561	33.2 (29.3–37.1)	23.5 (20.0–27.0)	19.8 (16.5–23.1)
Highly active overweight	947	25.3 (22.6–28.1)	16.8 (14.4–19.2)	15.5 (13.2–17.8)
Moderately active overweight	1185	31.3 (28.7–34.0)	21.4 (19.1–23.8)	19.4 (17.2–21.7)
Inactive overweight	881	33.5 (30.4–36.6)	23.7 (20.9–26.5)	20.3 (17.7–23.0)

In age and gender adjusted model the inactive normal-weight (HR 1.3, 95 % CI 1.1–1.5), moderately active overweight (HR 1.3, 95 % CI 1.1–1.5), and inactive overweight (HR 1.4, 95 % CI 1.2–1.6) had higher risk of psychotropic medication compared with the highly active normal-weight, however, the highly active overweight were not at increased risk (HR 1.0, 95 % CI 0.9–1.2) (Table 3). Adjusting for prior medication before baseline attenuated the associations after which only the inactive overweight group had a significantly higher risk (HR 1.4, 95 % CI 1.2–1.6) for any psychotropic medication. Adjusting for covariates in model 3 had only a small effect compared with model 2, but adjusting for LLI, smoking, and drinking problems (model 4) further attenuated the associations. However, the inactive overweight group still had a higher risk of psychotropic medication (HR 1.2, 95 % CI 1.0–1.4).

**Table 3:** Hazard ratios for psychotropic medication purchases according to joint association of physical activity and BMI

All	Model 1	Model 2	Model 3	Model 4
Highly active normal-weight	1.00	1.00	1.00	1.00
Moderately active normal-weight	1.17 (1.01–1.35)	1.15 (0.99–1.33)	1.15 (0.99–1.33)	1.09 (0.94–1.26)
Inactive normal-weight	1.28 (1.07–1.53)	1.21 (1.01–1.44)	1.20 (1.04–1.44)	1.15 (0.96–1.37)
Highly active overweight	1.01 (0.85–1.19)	1.00 (0.85–1.18)	1.01 (0.85–1.19)	0.96 (0.82–1.14)
Moderately active overweight	1.27 (1.10–1.48)	1.15 (0.99–1.34)	1.15 (0.99–1.34)	1.08 (0.93–1.26)
Inactive overweight	1.41 (1.20–1.65)	1.38 (1.18–1.62)	1.37 (1.17–1.61)	1.21 (1.04–1.42)
Antidepressants	Model 1	Model 2	Model 3	Model 4
Highly active normal-weight	1.00	1.00	1.00	1.00
Moderately active normal-weight	1.21 (1.01–1.45)	1.21 (1.01–1.44)	1.20 (1.00–1.43)	1.15 (0.97–1.38)
Inactive normal-weight	1.45 (1.17–1.79)	1.38 (1.12–1.71)	1.37 (1.11–1.67)	1.31 (1.06–1.63)
Highly active overweight	1.06 (0.86–1.29)	1.06 (0.87–1.30)	1.07 (0.87–1.31)	1.03 (0.84–1.25)
Moderately active overweight	1.39 (1.16–1.66)	1.25 (1.05–1.50)	1.26 (1.05–1.51)	1.19 (1.00–1.43)
Inactive overweight	1.59 (1.32–1.91)	1.55 (1.29–1.87)	1.53 (1.27–1.85)	1.36 (1.13–1.64)
Sedatives	Model 1	Model 2	Model 3	Model 4
Highly active normal-weight	1.00	1.00	1.00	1.00
Moderately active normal-weight	1.20 (1.00–1.44)	1.19 (0.99–1.43)	1.18 (0.99–1.42)	1.15 (0.95–1.38)
Inactive normal-weight	1.24 (0.99–1.56)	1.15 (0.91–1.44)	1.14 (0.91–1.43)	1.09 (0.87–1.37)
Highly active overweight	0.99 (0.80–1.21)	0.94 (0.76–1.16)	0.96 (0.78–1.18)	0.93 (0.76–1.15)
Moderately active overweight	1.23 (1.02–1.47)	1.12 (0.93–1.35)	1.13 (0.93–1.36)	1.05 (0.87–1.27)
Inactive overweight	1.32 (1.08–1.61)	1.28 (1.05–1.56)	1.27 (1.04–1.55)	1.14 (0.93–1.39)

Model 1 age and gender

Model 2 age, gender, and prior medication

Model 3 age, gender, prior medication, socioeconomic position, marital status, work mental and physical strenuousness

Model 4 age, gender, prior medication, limiting long-standing illness, drinking problems, and smoking

For antidepressant medication the associations were similar although slightly stronger than for the any psychotropic medication. After adjustment for prior medication the moderately active overweight, inactive normal-weight and the moderately active and inactive overweight groups had increased risk of antidepressant medication. After adjustment for baseline smoking, drinking problems and LLI (model 4), those physically inactive normal-weight (HR 1.3, 95 % CI 1.1–1.6) and overweight (HR 1.4, 95 % CI 1.1–1.6) had a higher risk of antidepressant medication.

For sedative medication the association was weaker than for antidepressants. Overweight was associated with increased risk of sedative medication among physically inactive (HR 1.3, 95 % CI 1.1–1.6) and moderately active (HR 1.2, 95 % CI 1.0–1.5) after adjusting for age and gender. After adjusting for prior medication the association remained only for the inactive overweight group. After full adjustments (model 4) no associations remained.

## Discussion

In this study we examined joint-association of physical activity and overweight with subsequent psychotropic medication over a follow-up of around 8 years.

Main findings were as follows:

1. Both normal-weight and physical activity are associated with lower risk of psychotropic medication but physical activity dominates the association over normal-weight.
2. Joint association of physical activity and overweight were stronger with antidepressants than sedatives

The results of our study confirm results from previous longitudinal [19, 33] and cross-sectional [20] studies with self-reported measures of mental health. We conducted the analyses also for antidepressants and sedatives, which are the two main groups of psychotropic medication. Associations were quite similar to any psychotropic medication but antidepressants had a stronger association than sedatives. Unfortunately, we do not have information on which mental disorder the medication was prescribed for. There is comorbidity between depression and anxiety [34] and antidepressants are also prescribed for anxiety disorders [35]. However, previous studies on physical activity and mental health problems have shown especially an association with depression [17]. We also conducted the analyses excluding those with both antidepressant and sedative purchases and it made a minor contribution to the examined associations.

Overall, the associations in this study were relatively weak but still relevant from public health perspective. Mental health problems are commonly occurring [1] and promoting physical activity among middle-aged employees may be useful for preventing mental health problems. Association between overweight or obesity and mental health problems are reciprocal [36] and in our study we only examined the obesity to mental health pathway. Although our study showed that overweight is unlikely to notably affect the risk of mental health problems, normal-weight has plenty of other considerable benefits for health such as better physical functioning.

A recent prospective study from United States [33] showed that cardiorespiratory fitness is more important than fatness in reducing depressive symptoms. Increasing physical activity is the only way to improve cardiorespiratory fitness thus those highly active in our study likely have better fitness than their less active counterparts which may also contribute to the differences found in psychotropic medication purchases. Previous review [7], however, concluded that even low doses of physical activity are beneficial in terms of depressive symptoms, supporting the view that there are positive effects of physical activity independent of fitness on mental health. Physical activity increases e.g. several neurotransmitters such as serotonin and dopamine which may protect especially against depression [37].

Mental health problems may also contribute to physical inactivity, and in addition, some psychotropic medication tends to increase body weight [38]. We were interested in joint association between physical activity and overweight with psychotropic medication in a follow-up and therefore we excluded those with baseline psychotropic medication and adjusted for previous medication in the analyses. We conducted the analyses excluding those with prior psychotropic medication within the last three years before the baseline survey. The analyses showed that the association were similar to those with previous medication adjusted for (data not shown). Furthermore, those with drinking problems have more anxiety and depression symptoms [39] and economic difficulties and socioeconomic position are also associated with mental health problems [40]. We adjusted these as well as other key covariates, however, more detailed control analyses showed that LLI attenuated the association the most. Some LLI may restrict physical activity and also increase the body weight and further contribute to mental health problems.

Additional sensitivity analyses were made. We used different cut-off points for body mass index and physical activity. We examined obesity (30 kg/m^2^) and also classified BMI groups as well as physical activity according to tertiles. Different cut-off points made a minor contribution to the examined associations suggesting that the associations are not sensitive to the cut-off points used (data not shown). In addition, we used defined daily dose (DDD) of 365 as outcome reflecting more severe problems in mental health. Thirty-six percent of those having any psychotropic medication had DDD over 365. We also divided psychotropic medication purchases to three categories: no purchases, 1 to 2 purchases, and 3 or more purchases. Sixty-three percent of those having any psychotropic medication had three or more purchases. However, these additional analyses with more severe outcomes showed similar associations with the first purchase as the outcome (data not shown).

The strengths of this study include a prospective design and a large sample of middle-aged women and men. Additionally, complete national register data of psychotropic medication purchases linked with the survey data, is strength. We excluded those with the baseline medication and adjusted for prior medication as well as several other covariates.

The limitations of this study include self-reported body mass index and leisure-time physical activity. People tend to overestimate physical activity and height and underestimate weight [41]. Despite of underestimation, self-reported BMI is an adequate predictor of associated health risks [42]. BMI cannot distinguish whether the mass is from fat or from muscle but in middle-aged population dominated by women this is likely not a major concern. In leisure-time physical activity we used cut-off points based on recommendation. Self-estimated leisure-time physical activity was not validated, however, no single physical activity questionnaire has proved better than others for measurements of leisure-time physical activity [43]. The psychotropic medication purchases are not a direct measurement for mental health because they have also other indications such as epilepsy, pain, and sleep problems for several psychotropic medicines. Some people with mental health problems have no psychotropic medication and it is also unclear whether people use the medicines they have purchased. However, we were interested in the need of psychotropic medication rather than if the participants had used the medication purchased. The need of psychotropic medication is estimated when doctors decide to prescribe psychotropic medication. We used only baseline information of weight and leisure-time physical activity as well as covariates and all these variables could change during the follow-up. However, this is unlikely to result in overestimating of the associations. Because 78 % of participants were women and genders were combined in the analyses, the results are dominated by the women. However, the association were not significantly different between genders.

## Conclusions

Both normal-weight and physical activity may help prevent psychotropic medication but physical activity dominates the association. Higher amounts of physical activity may show further benefits for mental health. Promoting physical activity among middle-aged employees may prove useful for preventing mental health problems.

## Abbreviations

BMI: Body mass index
SD: Standard deviation
MET: Metabolic equivalent task
Kcal: Kilocalories
ATC: Anatomical therapeutic chemical
LLI: limiting long-standing illness
HR: Hazard ratio
CI: Confidence interval
SPSS: Statistical package for the social sciences
DDD: Defined daily dose
